# Myasthenia Gravis With Antibodies Against Muscle Specific Kinase: An Update on Clinical Features, Pathophysiology and Treatment

**DOI:** 10.3389/fnmol.2020.00159

**Published:** 2020-09-02

**Authors:** Michelangelo Cao, Inga Koneczny, Angela Vincent

**Affiliations:** ^1^Nuffield Department of Clinical Neurosciences, Weatherall Institute of Molecular Medicine, University of Oxford, Oxford, United Kingdom; ^2^Division of Neuropathology and Neurochemistry, Department of Neurology, Medical University of Vienna, Vienna, Austria

**Keywords:** myasthenia gravis, MuSK, autoantibodies, neuromuscular junction, acetylcholine receptor

## Abstract

Muscle Specific Kinase myasthenia gravis (MuSK-MG) is an autoimmune disease that impairs neuromuscular transmission leading to generalized muscle weakness. Compared to the more common myasthenia gravis with antibodies against the acetylcholine receptor (AChR), MuSK-MG affects mainly the bulbar and respiratory muscles, with more frequent and severe myasthenic crises. Treatments are usually less effective with the need for prolonged, high doses of steroids and other immunosuppressants to control symptoms. Under physiological condition, MuSK regulates a phosphorylation cascade which is fundamental for the development and maintenance of postsynaptic AChR clusters at the neuromuscular junction (NMJ). Agrin, secreted by the motor nerve terminal into the synaptic cleft, binds to low density lipoprotein receptor-related protein 4 (LRP4) which activates MuSK. In MuSK-MG, monovalent MuSK-IgG4 autoantibodies block MuSK-LRP4 interaction preventing MuSK activation and leading to the dispersal of AChR clusters. Lower levels of divalent MuSK IgG1, 2, and 3 antibody subclasses are also present but their contribution to the pathogenesis of the disease remains controversial. This review aims to provide a detailed update on the epidemiological and clinical features of MuSK-MG, focusing on the pathophysiological mechanisms and the latest indications regarding the efficacy and safety of different treatment options.

## Overview

Myasthenia gravis with antibodies against Muscle Specific Kinase myasthenia gravis (MuSK-MG) is an autoimmune disease that impairs transmission at the neuromuscular junction (NMJ), leading to generalized weakness and fatigability of skeletal muscles. MuSK-MG represents an important subgroup of autoimmune myasthenia affecting 5–70% of patients (depending on geographical location) who are negative for the more common antibodies against the acetylcholine receptor (AChR; Hoch et al., 2001; Vincent and Leite, 2005). Compared to the form with AChR antibodies, MuSK-MG differs in terms of epidemiology, clinical features, pathogenic mechanisms, and response to treatment. Autoantibodies, which are mainly of the monovalent IgG4 subclass, target and block the function of MuSK, a tyrosine kinase located on the muscle post-synaptic membrane, disrupting a finely tuned pathway that regulates the development and maintenance of high-density clusters of AChRs at the NMJ. IgG1, 2 and 3 MuSK antibodies are also shown *in vitro* to be potentially pathogenic but their mechanisms *in vivo* have not been identified.

The following review will describe the clinical and pathophysiological features of MuSK-MG, focusing on the effect of the autoantibodies on the AChR clustering pathway, and the main principles of diagnosis and treatment.

## Epidemiology

Several epidemiological studies have been carried out since MuSK antibodies were discovered in 2001 (Hoch et al., 2001) and the incidence of MuSK-MG appears to vary widely across countries (Vincent et al., 2008). Therefore, the general prevalence of the disease is difficult to estimate due to this variability. Mediterranean countries, especially Italy, Turkey, and Greece, present the highest frequency of MuSK-MG (Evoli et al., 2003; Tsiamalos et al., 2009). On the other hand, prevalence appears to be lower among those populations that live at northern latitudes (Niks et al., 2007; Vincent et al., 2008) and higher in people of Afro-American origin (Oh et al., 2009). Considering this particular ethnic-geographical distribution, together with the association with HLA class II DR14 DQ5 (Niks et al., 2006; Bartoccioni et al., 2009; Alahgholi-Hajibehzad et al., 2013), genetic background is likely to play a significant role in the etiology of MuSK-MG although other—and still undetermined—environmental factors are likely to be involved.

Association with thymus pathology (either thymic hyperplasia or thymoma) has only rarely been reported and this has a direct implication on therapeutic management of patients as thymectomy is not indicated in the current clinical practice (Marx et al., 2013; Clifford et al., 2019).

MuSK-MG affects predominantly women (up to 9:1 female:male ratio), especially young females in their third decade (Evoli et al., 2003; Guptill et al., 2011) and it is reported rarely in elders and children (Pasnoor et al., 2010; Skjei et al., 2013). This is in contrast with the distribution of AChR-MG in which an increase of incidence in older males has been observed over the last few decades (Carr et al., 2010).

## Clinical Features

Like other diseases that affect neuromuscular transmission, the main clinical characteristic of MuSK-MG is the fluctuating weakness and fatigability of the skeletal muscles, which improve with rest and worsen after exercise. However, a diagnosis of MuSK-MG can be challenging as clinical signs of the disease, and pattern of muscle involvement can differ in several ways from other forms of myasthenia. In particular, symptom fluctuation may be subtle and, therefore, go unnoticed. Moreover, proximal limb involvement can be mild or even absent while respiratory distress and other bulbar symptoms, such as difficulties in swallowing, chewing, and speaking, can be severe and rapidly progressive. The misinterpretation of these symptoms, which could support a diagnosis of a primary myopathy or motor neuron disorder (Huijbers et al., 2016), may contribute to a delay in the recognition and treatment of MuSK-MG patients.

Ocular manifestations, usually consistent with mild and symmetrical ophthalmoparesis and ptosis (drooping and weakness of the eyelid), were initially thought to be less prominent in MuSK-MG as they were reported to be the only symptom at onset in 36% of MuSK-MG cases compared to 60% of AChR-MG (Guptill et al., 2011). However, a recent retrospective analysis of Italian patients showed that the involvement of extra-ocular muscles was present in more than 96% of the population studied, with a frequency comparable to AChR-MG, and represents the first manifestation of the disease in almost 60% of cases (Evoli et al., 2017). On the other hand, the purely ocular disease is considered to be particularly rare—or at least underdiagnosed—as all the patients in both those studies evolved into generalized MG over time.

A highly specific clinical feature of MuSK-MG is the involvement of bulbar and respiratory muscles, which affects most of the patients and can be particularly severe. Patients usually experience variable degrees of dysarthria (often with nasal voice), dysphagia (mainly for fluids and associated sometimes with weight loss), dysphonia, and dyspnoea (Evoli, 2006; Ohta et al., 2007; Pasnoor et al., 2010; Guptill et al., 2011). Respiratory crises are particularly frequent (up to 35% of cases) representing a serious life-threatening event and are described more commonly compared to AChR-MG (Vincent et al., 2003; Deymeer et al., 2007). The weakness of neck and axial muscles is usually associated with bulbar symptoms and, in patients with a long history of severe disease, facial and tongue atrophy represents a common finding (Evoli et al., 2003; Farrugia et al., 2006b). When muscle atrophy occurs, it is clinically associated with non-fluctuating weakness, myopathic changes in the electrophysiology recordings, and fatty tissue infiltration at the muscle MRI scans.

## Pathophysiology of MuSK-MG

### The AChR Clustering Pathway

**Figure 1:**
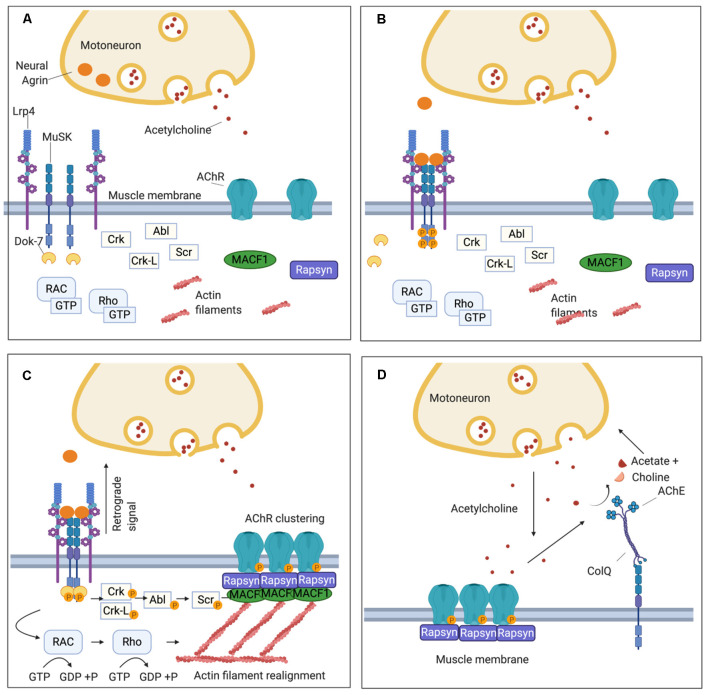
Schematic representation of the neuromuscular junction (NMJ) and the clustering pathway of acetylcholine receptors (AChRs). **(A)** On the muscle post-synaptic membrane, low density lipoprotein receptor-related protein 4 (LRP4) and Muscle Specific Kinase (MuSK) form a loose tetramer, and, at this stage, AChRs are dispersed along the sarcolemma. **(B)** Agrin is secreted by the motor nerve terminal into the synaptic cleft and interacts with LRP4 inducing a conformational change in the LRP4-MuSK tetramer. This leads to MuSK activation through an auto-phosphorylation loop of the tyrosine residues in its intracytoplasmatic domain. **(C)** The recruitment of DOK7 amplifies MuSK phosphorylation and triggers the phosphorylation cascade downstream that involves several intermediate proteins including Crk, Ckr-l, Abl, and Scr. Concomitantly, a subsidiary pathway leads to cytoskeletal rearrangements through the activation of Rac1 and Rho GTPases. The clustering pathway culminates in the phosphorylation of the subunits of the AChR and the recruitment of rapsyn, which anchors the AChR clusters to the underlying actin filaments. Furthermore, LRP4 participates in presynaptic development through a retrograde signal which increases the clustering of ACh vesicles in the motor nerve terminal. **(D)** *Via* the extracellular matrix (ECM), MuSK interacts also with ColQ which, in turn, is attached in tetramers to the enzyme acetylcholinesterase (AChE) responsible for the hydrolysis of acetylcholine (ACh) after its binding to the AChR.

To understand how autoantibodies directed against MuSK impair transmission at the NMJ, leading to the full clinical spectrum of myasthenia, it is necessary to understand the key function of MuSK in the development and maintenance of the NMJ and particularly in the clustering of AChRs (Figure 1). MuSK is essential during the early stages of NMJ development, mediating the signaling between the motor nerve terminal and the muscle fiber, and guiding innervation by the growing motor nerve towards areas where pre-patterned AChR clusters are expressed (DeChiara et al., 1996; Glass et al., 1996b; Flanagan-Steet et al., 2005; Panzer et al., 2006).

The signaling molecule that triggers all the downstream events of the AChR clustering pathway is a heparan-sulfate proteoglycan called agrin, which is secreted by the motor nerve terminal and binds to its receptor, low-density lipoprotein receptor-related protein 4 (LRP4), expressed on the muscular sarcolemma (Godfrey et al., 1984; McMahan, 1990; Glass et al., 1996a; Zhang et al., 2008; Figures 1A,B). LRP4 plays a double role in synapsis maturation: it mediates agrin-induced MuSK activation and, through a retrograde-signaling to the motor axon, it contributes to the pre-synaptic maturation increasing the clustering of acetylcholine vesicles (Wu et al., 2012; Yumoto et al., 2012; Figure 1C). Alongside agrin, laminin-121 and other proteins of the laminin family, which are synthesized by the muscle cell and expressed in the basal lamina, are essential in shaping the final structure of the NMJ. They contribute to the formation of the junctional folds and balance the growth of the pre-synaptic components of the NMJ such as the synaptic bouton of the nerve terminal and the Schwann cells (Noakes et al., 1995; Patton et al., 2001).

When agrin binds to the first β-propeller (BP) domain of LRP4 (Zhang et al., 2011), it strengthens a heteromeric tetramer with MuSK in a 2:2 stoichiometry. This conformational change in LRP4-MuSK interaction triggers the auto- and trans-phosphorylation of MuSK intracytoplasmic domain and the beginning of a phosphorylation cascade that culminates with the clustering of AChRs (Zong et al., 2012; Figure 1B). To sustain MuSK activation and stimulate the downstream pathway, however, the recruitment of an additional intracellular adaptor, the Downstream of Kinase 7 (DOK7), is necessary. DOK7, in the form of a dimer, interacts with the intracytoplasmic domain of MuSK, enhancing the trans-phosphorylation of MuSK’s tyrosine residues (Bergamin et al., 2010).

MuSK and DOK7 activate each other and this reciprocal activation triggers all the downstream events, which have been only partially defined. Following MuSK-DOK7 activation, several other adaptor proteins are phosphorylated and recruited, in particular Crk and Crk-L (Hallock et al., 2010), as well as other kinases, such as Abl (Finn et al., 2003), Scr (Mittaud et al., 2001), and the GTPases Rac1 and Rho which induce cytoskeleton modifications contributing to the maturation of AChR clusters (Weston et al., 2003; Bai et al., 2018). The whole cascade converges into the phosphorylation of the AChR subunits and rapsyn; rapsyn is a structural protein that self-aggregates providing a scaffold that anchors AChRs with the actin cytoskeleton—through the mediation of microtubule actin cross-linking factor 1 (MACF1)—to form the mature clusters (Borges and Ferns, 2001; Lee et al., 2009; Zuber and Unwin, 2013; Oury et al., 2019; Xing et al., 2019; Figure 1C).

To prevent the formation of AChR clusters in extrasynaptic sites, and to reduce the risk of tetanic contraction caused by an overstimulation of the muscle, several negative regulators control the clustering pathway and the expression of AChRs on the muscle membrane. One of the most important of these regulators is acetylcholine itself which downregulates the expression of AChRs. During neuromuscular transmission, ACh binds to the AChRs and subsequently is removed by acetylcholinesterase (AChE), within the extracellular matrix (ECM), that hydrolyzes acetylcholine to choline and acetate. AChE is bound to ColQ, the collagen that is arranged as trimers to form a triple helix. AChE binds to the collagen tails of ColQ in the form of tetramers (Krejci et al., 1991; Gašperšič et al., 1999). ColQ is located in the ECM, where it is anchored *via* MuSK to the synapse (Cartaud et al., 2004; Figure 1D).

In the absence of AChE, sustained ACh stimulation induces a prolonged Ca^++^ influx which activates calpain, a protease constitutively inhibited by rapsyn, triggering AChR cluster dispersal mediated by Cdk5 (Lin et al., 2005; Chen et al., 2007). While in AChR-MG an excess of ACh caused by treatment with AChE inhibitors does not contribute significantly to AChR loss, perhaps because it is effectively counterbalanced by the activation of a functional MuSK-DOK7 pathway, AChE inhibitors exacerbate MuSK-MG symptoms; this is likely due to a downregulation of the AChR following the ACh increase without the counterbalancing provided by the MuSK pathway (see the following section regarding treatment). Also, the prolongation of endplate currents observed in a MuSK-MG mouse model (Patel et al., 2014) suggests that MuSK antibodies that interfere with the interaction between MuSK and ColQ (Kawakami et al., 2011; to which AChE is attached) might reduce the catalytic activity of the AChE further contributing to the resistance of MuSK-MG patients to treatment with AChE inhibitor (Patel et al., 2014).

Another negative regulator of the clustering pathway is the SRC homology two domain-containing phosphotyrosine phosphatase 2 (SHP2). This phosphatase is activated by MuSK through the intermediate action of Src and SIRPα1 proteins and, in a negative feedback loop, it reduces MuSK phosphorylation (Zhao et al., 2007). SHP2 is thought to also play a role during embryonic development by preventing the formation of AChR clusters in extra-synaptic sites that are not directly in contact with the motor nerve terminal (Qian et al., 2008). From a clinical perspective, targeting this or other negative regulators of AChR clustering could represent a potential strategy to develop novel and specific therapeutic approaches.

### MuSK Antibodies—Discovery and Pathogenicity Studies

The constant and finely-tuned activation of the agrin-LRP4-MuSK-DOK7 pathway maintains the AChRs in high-density clusters on the post-synaptic membrane that is vital for the correct function of the NMJ. Indeed, clustered receptors are optimal to respond to the signal transmitted by the motor nerve terminal, channeling the depolarization down into the folds where the opening of the voltage-gated sodium channels triggers the muscle action potential.

The existence of a distinct target for patients with MG who did not have AChR antibodies had been proposed as early as 1976 (Lindstrom et al., 1976) and demonstrated, in principle, in 1986 in which immunoglobulin preparations from AChR-Ab negative myasthenic patients were able to impair neuromuscular function (Mossman et al., 1986). At that time, a specific target was not identified but, retrospectively, more than half the patients used were positive for MuSK antibodies (AV unpublished data). Eventually, the existence of an IgG antibody was shown by demonstrating the binding of AChR-Ab negative patient IgG to the TE671 human immortalized muscle cells (Blaes et al., 2000; Scuderi et al., 2002). In 1996, the Yancopoulos group at Regerneron Inc USA had discovered MuSK as an essential protein in muscle development restricted to the NMJ (Glass et al., 1996a). MuSK was thus a very good candidate for the missing target and this was demonstrated by showing binding to COS cells expressing recombinant MuSK, and by binding to the recombinant extracellular domain of MuSK in an ELISA. Also, MuSK IgG antibodies inhibited agrin-induced AChR clustering on C2C12 myotubes (Hoch et al., 2001). MuSK was shown independently to be immunoprecipitated and identified by mass spectroscopy by the Evoli group (Scuderi et al., 2002) and the approach to immunoprecipitate MuSK from the TE671 cells confirmed and optimized by Littleton et al. (2009).

**Figure 2:**
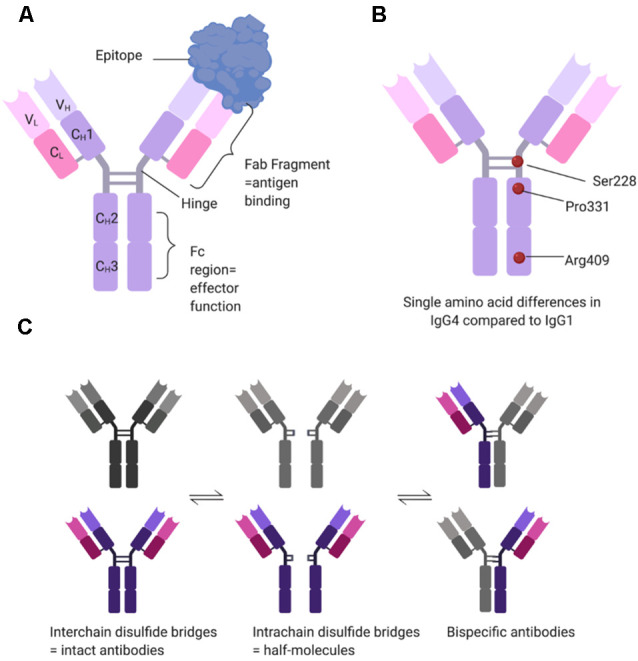
Structural determinants of IgG4 subclass antibodies. **(A)** IgG4 consists of two heavy (H) and two light (L) chains, each with one variable (V) domain, and one (CL) or three (CH1–CH3) constant domains. In the hinge region, the two heavy chains are connected covalently by two interchain disulfide bridges. The variable domains and the first constant domain form the Fab fragment, which binds the antigen, while the hinge and the CH2–CH3 domains form the Fc region which provides effector function such as binding of C1q or Fc gamma receptors. **(B)** IgG1 and IgG4 have over 90% sequence homology, but single amino acid differences affect the structure and function of the antibody. Three relevant examples are serine at position 228 instead of a proline, allowing for structural flexibility and a switch to intrachain disulfide bridges. Proline at position 331 prevents binding of C1q, and arginine at position 409 leads to reduced non-covalent interactions between the two heavy chains. **(C)** Under reducing conditions (e.g., 1 mM glutathione), IgG4 can undergo Fab-arm exchange. The interchain disulfide bridges change to intrachain disulfide bridges, disconnecting the two half-molecules of the antibody. These then stochastically recombine with other half-molecules, forming bi-specific antibodies. Under reducing conditions, the different states of IgG4 are in equilibrium.

One of the most intriguing aspects of the pathogenicity of MuSK antibodies resides within the nature of the antibodies themselves. Compared to AChR-MG, where autoantibodies are mainly of the IgG1 and less frequently IgG3 subclasses (Vincent and Newsom-Davis, 1982), MuSK antibodies are, on the contrary, mostly IgG4s (McConville et al., 2004). Unlike IgG1-3 antibodies, IgG4s are functionally monovalent (Figure 2). This is because single amino acid differences between IgG1 and IgG4 in the hinge region of the CH3 backbone allow for greater stereometric flexibility (Figures 2A,B). As a consequence, intrachain (instead of interchain) disulfide bridges form under reducing conditions, thereby leaving the two half-molecules of IgG4 without covalent connection (van der Neut Kolfschoten et al., 2007). Further single amino acid differences in the CH3 domain reduce noncovalent interactions between the heavy chains (Figure 2B). Taken together this allows the Fab-arm exchange phenomenon (reviewed in Vidarsson et al., 2014) by which each half-antibody exchanges with other half-antibodies that are randomly selected from the pool of total IgG4 half-antibodies (Figure 2C). As a result, most if not all IgG4 MuSK antibodies are monovalent, rather than divalent, for their antigen (Koneczny et al., 2017), which does not allow for divalent-dependent internalization of the antigen, a mechanism widely recognized in antibody-mediated diseases. Additional single amino acid differences in the CH2 domain of IgG4 render it unable to bind to C1q to activate the complement cascade or FcγR to activate immune cells. Instead, IgG4 antibodies can only block the function of a protein or inhibit its interaction with other proteins (reviewed in Koneczny, 2018).

With the discovery of MuSK as the antigen, both *in vitro* and *in vivo* models provided strong evidence of the pathogenicity of MuSK antibodies. Subsequent experiments carried out on different animal models—either passively immunized with MG patients’ plasma or actively immunized with purified recombinant MuSK—showed clinical symptoms and/or impaired neuromuscular transmission consistent with the myasthenic phenotype, including muscle weakness and fatigability, reduction of endplate AChR numbers, and reduction of endplate potential (EPP) and miniature EPP amplitudes (Shigemoto et al., 2006; Cole et al., 2010; Klooster et al., 2012; Viegas et al., 2012). Notably, delayed-synapsing muscles (specifically diaphragm, sternomastoid and tibialis posterior), in which synaptogenesis requires a longer period to complete, were more severely affected in a MuSK immunized model compared to fast-synapsing muscles (intercostal, adductor longus and tibialis anterior; Xu et al., 2006). This suggests that differences in muscle development could affect susceptibility to the effects of MuSK antibodies and might partially explain the clinical pattern of weakness in MuSK-MG.

Insights regarding the pathogenic mechanism by which MuSK antibodies cause myasthenia were provided by further *in vitro* experiments performed on cultured myotubes, which are derived from the mouse C2C12 immortalized muscle cell line. In mouse myotubes incubated with plasma or purified antibodies obtained from MuSK-MG patients, MuSK phosphorylation was markedly reduced (Huijbers et al., 2013; Koneczny et al., 2013); and not restored by recombinant agrin. Moreover, AChR clustering was substantially reduced (Hoch et al., 2001; Koneczny et al., 2013; Huda et al., 2020). The inhibition of MuSK phosphorylation was shown to be dependent on IgG4 antibodies, which prevented the binding of LRP4 to MuSK (Huijbers et al., 2013; Koneczny et al., 2013).

**Figure 3:**
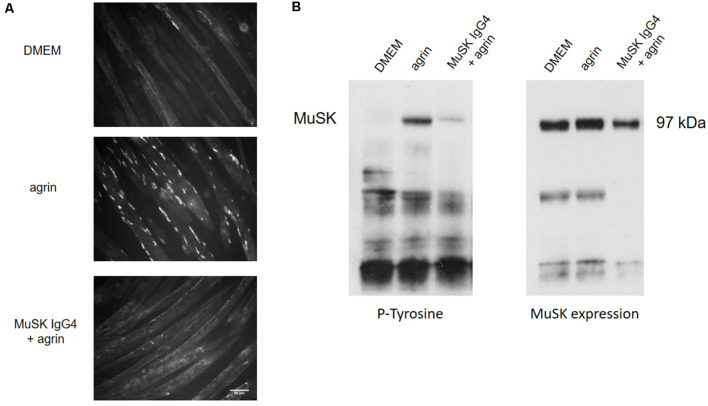
Effects of MuSK IgG4 antibodies on AChR clustering and MuSK phosphorylation on C2C12 mouse myotubes. **(A)** Myotubes form few spontaneous AChR clusters (upper panel—DMEM) but, after physiological stimulation with agrin, their number increase markedly (middle panel—agrin). However, when the myotubes are exposed to MuSK antibodies, agrin-induced AChR clustering is severely impaired (lower panel—MuSK IgG4). Images are taken with an Olympus IX71 fluorescence microscope at 20× magnifications. Scale bar represents 50 μm. **(B)** Example of western blots to analyze MuSK phosphorylation. On the left, blot probed for phosphorylated tyrosine residues; on the right, the same blot is stripped and reprobed for MuSK expression. MuSK corresponds to a band at 97 kDa. MuSK phosphorylation is typically not detectable in the presence of medium only (first column—DMEM). After incubation with agrin for 45 min, MuSK phosphorylation increases markedly (second column—agrin) but this is prevented in the presence of MuSK IgG4s (third column—MuSK IgG4). Unpublished work, similar to Koneczny et al. (2013) and Huda et al. (2020).

Figure 3 shows an example of the *in vitro* effect of MuSK purified IgG4 antibodies on C2C12 myotubes for AChR clustering and MuSK phosphorylation. In this established model, to study the clustering of AChRs, myotubes are exposed to the compound/sample of interest (such as recombinant agrin, patients’ plasmas, or purified antibody preparations) up to 16 h to allow full cluster maturation. The AChR clusters are then labeled with fluorescent α–bungarotoxin, a toxin purified from the krait snake venom that binds specifically and irreversibly to the α subunit of the AChR (Changeux et al., 1970). The shape, size, and number of AChR clusters are then analyzed and quantified. Typically, myotubes express few spontaneous AChR clusters (Figure 3A—DMEM). After overnight incubation with agrin, the number of clusters dramatically increases (Figure 3A—agrin) but this is prevented by the presence of MuSK antibodies (Figure 3C—MuSK IgG4 + agrin). Alongside the analysis of AChR clustering, MuSK phosphorylation can be detected through western blotting after incubation of the cells with the antibodies and immunoprecipitation of MuSK. A representative western blot is shown in Figure 3B. The western blot membrane is first probed with an anti-phosphotyrosine antibody and the bands corresponding to MuSK identified at 97 kDa (Figure 3B, left). The level of phosphorylation is then normalized to the total expression of MuSK which is detected on the same nitrocellulose membrane that has been stripped and reprobed with a specific anti-MuSK antibody (Figure 3B, right). Basal MuSK phosphorylation is usually barely detectable in normal myotubes but increases sharply after agrin incubation (bands shown in Figure 3B are after 45 min of exposure). Consistently with the inhibition of AChR clustering, MuSK-IgG4 antibodies prevent the increase of MuSK phosphorylation in the presence of normal agrin stimulation.

**Figure 4:**
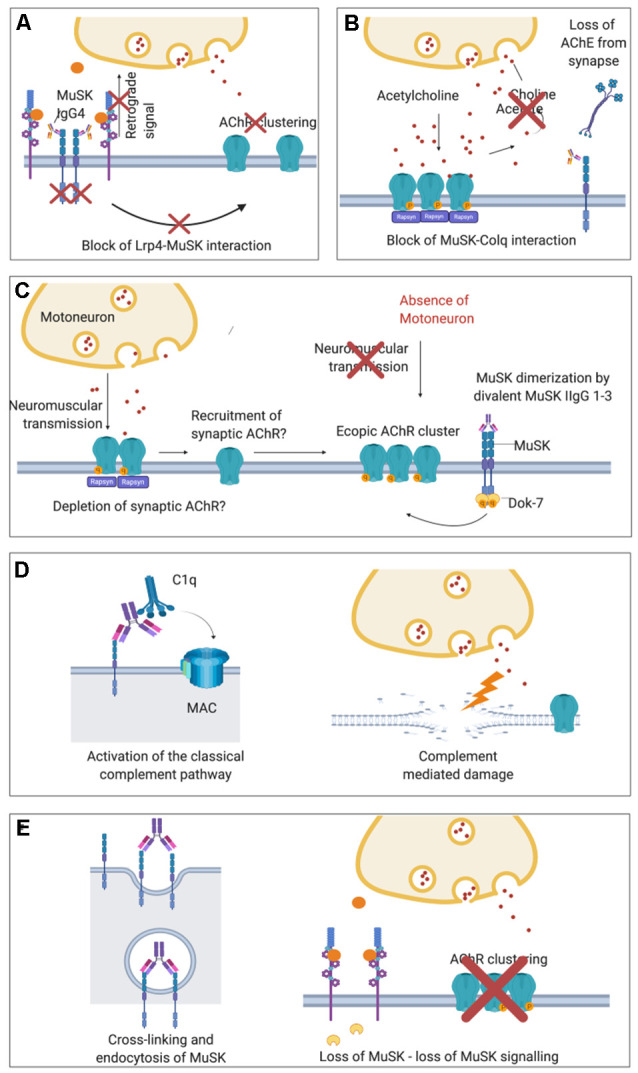
Proposed pathological mechanisms of MuSK autoantibodies at the NMJ. **(A)** In MuSK-MG, autoantibodies of the IgG4 subclass bind to the ectodomain of MuSK preventing its interaction with LRP4 and therefore block down-stream signaling. The phosphorylation cascade is therefore inhibited causing the ultimate dispersal of AChR clusters. Loss of retrograde signaling mediated by LRP4 may also explain the absence of presynaptic adaptive increase of ACh vesicles observed in MuSK-MG animal models which are otherwise characteristic in AChR-MG. **(B)** Block of ColQ-MuSK binding by MuSK autoantibodies has been proposed to be a pathogenic mechanism that may lead to a loss of AChE from the ECM at the NMJ. **(C)** Divalent binding of commercial or cloned (monospecific) MuSK antibodies has been proposed to cause MuSK dimerization and activation, leading to ectopic, extra-synaptic AChR clusters. These would not participate in neuromuscular transmission as they would lack adjacent motoneuron terminals. For the formation of ectopic AChR clusters, AChR may also be recruited from synapses thus depleting the NMJ from AChR. **(D)** *In vivo* MuSK antibodies (IgG1–3) may recruit C1q and activate the classical complement pathway, causing complement-mediated damage at the NMJ. **(E)** MuSK antibodies could also cross-link MuSK and lead to increased internalization of MuSK, thus effectively depleting MuSK from the muscle membrane and cause a loss of agrin-LRP4-MuSK-DOK7 signal transduction.

MuSK antibodies were demonstrated to prevent the interaction between LRP4 and MuSK (Koneczny et al., 2013; Figure 4A). They target mainly the first Ig-like domain of MuSK, an epitope which is located on the extracellular part of the protein and mediates LRP4-MuSK binding (McConville et al., 2004; Otsuka et al., 2015). According to this pathogenic model, the disruption of the functional tetramer formed by MuSK and LRP4 prevents MuSK from being activated following agrin-LRP4 binding (Huijbers et al., 2013; Küçükerden et al., 2016). MuSK is therefore not able to respond to agrin stimulation and, consequently, the entire AChR clustering cascade is inhibited. The on-going turnover of AChRs and dispersal by acetylcholine (see above) leads ultimately to the reduction in AChR clusters on the post-synaptic membrane of the NMJ. Moreover, *in vivo* models of MuSK-MG lack the presynaptic adaptive increase of ACh release (quantal content) observed in AChR-MG models (Mori et al., 2012; Viegas et al., 2012; Patel et al., 2014). A likely explanation is that MuSK antibodies disrupt the retrograde signaling mediated by LRP4, which increases the release of acetylcholine compensating the dispersal of AChRs (Figure 4A). An additional proposed mechanism of MuSK antibodies, that could be available to both IgG 1–3 and monovalent IgG4 autoantibodies, could be to block the ColQ-MuSK interaction (Figure 4B; Kawakami et al., 2011). This could reduce AChE at the MuSK-MG synapse and, conversely, increase the concentration of ACh within the synapse and causing dispersal of the receptors; it is proposed that this could be responsible for the hypersensitivity to treatment with AChE inhibitors in MuSK MG patients (Evoli et al., 2008; Morsch et al., 2013).

### Another Player of the Game: The IgG1, 2 and 3 Subclass Antibodies

Alongside the prominent, and better-characterized, MuSK IgG4 antibody population, a variable proportion of MuSK IgG1, 2 and 3 antibodies are usually detectable in most patients. The role and importance of these antibody subclasses are still a matter of debate but there is evidence that IgG1-3s could also actively contribute to the pathogenic process of MuSK-MG. Similar to IgG4s, MuSK IgG1-3s are indeed able to disperse *in vitro* agrin-induced AChR clusters in C2C12 myotubes, even though they do not inhibit the LRP4-MuSK interaction (Koneczny et al., 2013). In the same study, IgG1-3s also prevented cluster formation in an additional *in vitro* model in which AChR clusters were constitutively induced in the myotubes by the overexpression of *DOK7* rather than by the normal stimulation with agrin. As DOK7 acts downstream of MuSK, these findings suggest that the effect of MuSK IgG1-3s may not be limited to the potential disruption of LRP4-MuSK-DOK7 interaction but could also involve other parts of the AChR clustering pathway.

In addition to these findings, *in vivo* evidence of IgG1-3 pathogenicity is provided by a model in which a mouse knockout of murine IgG1-equivalent to human IgG4-developed myasthenic features when immunized against MuSK. In this case, the immune response was sustained by the murine equivalent of human IgG1-3 antibodies (Küçükerden et al., 2016) and the authors suggested that both complement activation and direct blocking of LRP4-MuSK interaction could be the underlying mechanisms of the disease.

Initial insights on the pathogenic mechanism through which MuSK IgG1-3s might act is suggested by two recent *in vitro* studies in which monoclonal MuSK antibodies were generated from patient-derived clonal MuSK-specific B cells and plasma cells and tested on C2C12 myotubes (Huijbers et al., 2019; Takata et al., 2019). These antibodies were engineered and produced as divalent proteins irrespective of their original subclasses (either IgG1, 2 or 3 or IgG4). In the absence of physiological agrin stimulation, these monoclonal antibodies actively increased MuSK phosphorylation (likely due to their divalent binding to two MuSK molecules) and, at the same time, AChR clustering was inhibited. Interestingly, divalent MuSK antibodies were found to induce low levels of AChR clustering, which were suggested to result in extra-synaptic AChR clusters that may deplete AChR from synapses (Figure 4C). Nevertheless, the number of induced AChR clusters was very low. Mutations in the kinase domain of *MUSK* which increases MuSK phosphorylation while impairing the clustering of AChRs have also been reported recently (Rodríguez Cruz et al., 2020) suggesting that a non-physiological MuSK activation could be detrimental for the formation of clusters. Since gain-of-function mutations and MuSK-activating antibodies both impair AChR clustering, there are new and still unanswered questions regarding the precise mechanisms through which MuSK phosphorylation regulates the clustering pathway. Further potential mechanisms that have been suggested for MuSK IgG1-3s are activation of complement (Figure 4D; Tüzün et al., 2011) and cross-linking and endocytosis of MuSK (Figure 4E; Cole et al., 2010). These are unlikely to be mechanisms of IgG4s, as it cannot bind C1q and activate complement, and is bi-specific and unable to cross-link MuSK. The mechanisms are, however, in theory, available to MuSK IgG1-3 subclass antibodies.

## Diagnosis of MuSK-MG

As for other disorders of the NMJ, the diagnosis of MuSK-MG is based on clinical symptomatology, electromyography recording, and antibody detection. Clinical suspicion arises when the patient presents with typical myasthenic features, namely fluctuating muscle weakness and fatigability, diplopia and/or eyelid ptosis, and predominant involvement of bulbar and respiratory muscles. Additional empirical evidence that supports the diagnosis of MuSK-MG is the lack of response—or even the worsening of symptoms—following administration of AChE inhibitors (Evoli et al., 2003; Guptill et al., 2011). Although the exact mechanism that sustains this effect is not completely understood, *in vivo* studies showed that MuSK-MG models lack the presynaptic adaptive increase of ACh release observed in AChR-Ab models (Mori et al., 2012; Viegas et al., 2012; Patel et al., 2014). Furthermore, a loss of AChE from the synapse as a consequence of a block of ColQ-MuSK interaction has been proposed (Kawakami et al., 2011). Following treatment with AChE inhibitors, the higher amount of ACh released into the synaptic cleft could increase the physiological dispersal of AChRs, which in turn would not be compensated by a functional agrin-LRP4-MuSK activation, therefore worsening the condition (Morsch et al., 2013).

When performed on clinically affected muscles (usually face and neck), electrophysiological studies with decrement and/or single-fiber jitter recording represent a useful tool to support the diagnosis of myasthenia, while studies on limb muscles may be negative (Farrugia et al., 2006a). Nevertheless, particular attention must be taken in the interpretation of electrophysiology results in cases of suspected MuSK-MG as misleading signs of denervation and neuromyotonia has been reported in the presence of MuSK antibodies (Simon et al., 2013; Furuta et al., 2015; Huijbers et al., 2016). Therefore, the whole clinical picture of the patient should always be evaluated carefully.

**Figure 5:**
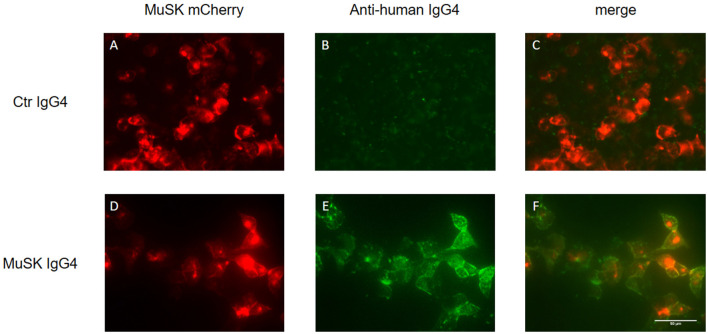
Cell-based assay (CBA) of MuSK IgG4 antibody subclasses. HEK293 cells are transfected with MuSK-mCherry (in red; **A,D**) and exposed to MuSK-MG IgG4 antibodies (lower panels) or antibodies purified from a healthy individual as control (upper panel). MuSK antibodies attached to the cell surface are then detected with a secondary fluorescent antibody specific for the IgG subclasses of interest (in green; **B,E**). The merged image from healthy control shows the absence of co-localization between MuSK-transfected HEK cells and purified antibodies **(C)**. Conversely, MuSK IgG4 antibodies give a very strong positive signal **(E)** with full colocalization with MuSK in the merge picture **(F)**. Images are taken with an Olympus IX71 fluorescence microscope at 40× magnifications. Scale bar represents 50 μm. Unpublished work, similar to Huda et al. (2017).

The detection of MuSK antibodies in patients’ sera represents the gold standard to confirm a clinical diagnosis of MuSK-MG. Three different laboratory techniques are available to detect MuSK antibodies: the radioimmunoprecipitation assay (RIA), ELISA, and the cell-based assay (CBA). The RIA represents the most common and specific test, reaching almost 100% of specificity (although it is difficult to estimate its sensitivity due to the variability in the proportion of MuSK-MG patients among the different populations studied). The assay involves immunoprecipitation of the extracellular domain of ^125^I-MuSK incubated with patient sera (Matthews et al., 2004; McConville et al., 2004). MuSK ELISAs are commercially available and occasionally used in research studies, but we observed that a small fraction of MuSK antibody-positive patient sera tested by RIA was not recognized as positive in ELISA (Koneczny et al., 2017). Alternatively, a CBA can be used, avoiding the need for radioactivity and providing a more physiological environment of the antigen, as MuSK is expressed in mammalian cells, warranting appropriate glycosylation, folding and positioning at the cell surface. A typical CBA is shown in Figure 5 in which HEK 293 cells are transfected with recombinant MuSK tagged with a fluorescent protein (in this case mCherry) and then exposed to different dilutions of serum from MuSK-MG patients and healthy individuals (used as control). MuSK antibodies attached to the cell surface are then detected with a secondary fluorescent anti-human antibody. Positivity is graded according to the degree of cell surface fluorescence and co-localization with labeled MuSK-transfected cells (Leite et al., 2008; Huda et al., 2017). The use of the CBA on patients who were seronegative by RIA—for both AChR and MuSK antibodies—slightly increased MuSK antibody detection. On those patients detected only with the MuSK CBA, it is worth noting that myasthenic symptoms were reported to be milder and their antibodies were less effective in inhibiting AChR clustering in *in vitro* assays (Huda et al., 2017).

## Treatment of MuSK-MG

In general terms, myasthenia gravis can be considered one of the few treatable neurological disorders in which a complete and stable remission can be achieved for a significant proportion of patients. However, the form caused by antibodies against MuSK often represents a difficult therapeutic challenge due to its severity and often poor response to treatment.

### Symptomatic Drugs

As mentioned above, symptomatic therapy with acetylcholinesterase inhibitors is often ineffective and, conversely, can induce exacerbation of myasthenia (Evoli et al., 2003; Guptill et al., 2011). Another symptomatic drug, 3,4-diaminopyridine that improves neuromuscular transmission by increasing the presynaptic release of ACh vesicles, showed promising results when tested in an *in vivo* model (Morsch et al., 2013) and when administered on a few selected pediatric and adult MuSK-MG patients (Skjei et al., 2013; Evoli et al., 2016). Increasing the release of ACh vesicles without affecting the duration of ACh in the synaptic cleft could be one reason why the 3,4-diaminopyridine is more effective than ACh-esterase inhibitors. The β-adrenergic agonists salbutamol and ephedrine have been successfully used in the treatment of congenital myasthenic syndromes, specifically with mutations in *AGRN*, *DOK7, COLQ*, and *MUSK* (Lee et al., 2018; Owen et al., 2018), and also showed effectiveness when tested on a MuSK-MG patient (Haran et al., 2013). In addition to the evidence provided by empirical clinical experience, the use of sympathomimetic drugs in myasthenic syndromes is supported by evidence regarding the role of sympathetic innervation in maintaining shape, size, and function of NMJs (Khan et al., 2016). In this study, chemical sympathectomy induced a reduction in size and complexity of the NMJs, and a concomitant decrease in amplitude and time to peak of the compound muscle action potentials (CMAP) in model animals. These effects were rescued by sympathomimetics. Although this treatment approach appears to be promising, randomized clinical trials are still needed to evaluate systematically the potential efficacy, and safety, in MuSK-MG.

Finally, it is worth mentioning that counteracting the specific pathogenic effects of IgG4 antibodies on MuSK phosphorylation could represent a novel approach in the development of new symptomatic treatments for MuSK-MG. A recent *in vitro* study showed that the selective inhibition of an intracellular phosphatase, the SH2 domain-containing phosphatase 2 (SHP2), can increase MuSK phosphorylation and prevent AChR clusters from dispersal when C2C12 myotubes were exposed to MuSK IgG4 antibodies, preventing their detrimental effects (Huda et al., 2020). A similar approach to preserve the integrity of the NMJ was performed in *SOD1-G93A* mice, a model of motor neuron disease, which were treated with a monoclonal antibody to MuSK that directly increases MuSK phosphorylation (Cantor et al., 2018). In this case, boosting the activation of MuSK slowed the course of the experimental disease, preventing the progression of diaphragm denervation, and prolonged the overall lifespan of the animals. Although different models and experimental techniques were used in the two aforementioned studies, their results suggest that modulating MuSK or downstream phosphorylation could represent a promising way towards the development of new selective treatments for disorders affecting the NMJ.

### Corticosteroids and Other Immunomodulatory Treatments

Overall, the general guidelines for the use of steroids and other immunosuppressants do not differ between MuSK- and AChR-MG, and treatment should be tailored according to the individual response of each patient. Steroids, in particular prednisone and prednisolone, are generally introduced early on for their effectiveness in controlling symptoms. Response to steroids is variable and, compared to AChR-MG, a higher proportion of MuSK-MG patients require high doses and prolonged treatment to achieve full control of the disease (Guptill and Sanders, 2010; Evoli et al., 2012). The major issue of long-term administration of steroids concerns the occurrence of relevant side effects such as increased blood pressure, risk of diabetes, overweight, osteoporosis, and possibly increasing a tendency to muscle atrophy already significant in MuSK-MG patients (Benveniste et al., 2005; Farrugia et al., 2006b). For these reasons, once full symptom control is reached, the steroids should be decreased progressively to achieve the lowest dose that maintains pharmacological remission. Immunosuppressive drugs, such as azathioprine and mycophenolate, are often introduced as steroid-sparing agents when it is not possible to wean the patient from a long-term high dose of steroids. However, a high proportion of MuSK-MG patients require combined therapy with immunosuppressants and steroids to achieve satisfactory symptom control, further highlighting an overall lower response to treatments compared to AChR-MG (Evoli et al., 2008, 2012; Sanders and Evoli, 2010).

The treatment of severe relapses, with mainly bulbar symptomatology, and life-threatening myasthenic crisis are similar between MuSK- and AChR-MG. Acute administration of intravenous immunoglobulins (IVIg) and plasma exchange are both usually effective (for the latter up to 96% of positive response irrespective of patients’ antibody status) with rapid control of the symptoms which last for about 4–6 weeks (Usmani et al., 2019). Although considered equal in the treatment of myasthenia (Rønager et al., 2001), plasma exchange has been reported to be faster and more effective compared to IVIg in MuSK-MG patients (Guptill and Sanders, 2010; Pasnoor et al., 2010) and, therefore, should be prioritized.

Refractory MG patients, in whom at least two independent immunosuppressive treatments have been carried out at therapeutic dosage without benefit in controlling the disease, should be considered for treatment with rituximab, a monoclonal chimeric IgG1 that depletes B lymphocytes through specific binding to the CD20 transmembrane antigen (Maddison et al., 2011; Kaegi et al., 2019; Di Stefano et al., 2020). In MuSK-MG patients, rituximab treatment appears to be particularly effective and this could be because IgG4 antibodies sustain the main pathogenic mechanism of the disease. Short-lived plasma cells producing IgG4 antibodies may be more susceptible to the drug as indicated by studies on other diseases caused by IgG4 antibodies (such as pemphigus and IgG4-related disease) in which significant clinical improvement and reduction in antibody titers were observed (Khosroshahi et al., 2010; Díaz-Manera et al., 2012; Carruthers et al., 2015; Kamisawa and Okazaki, 2017; Kurihara et al., 2019). The use of rituximab treatment in MuSK-MG is supported by several case reports, local studies, and, more recently, by bigger nation-wide group analysis (Hain et al., 2006; Khosroshahi et al., 2010; Nowak et al., 2011; Díaz-Manera et al., 2012; Keung et al., 2013; Hehir et al., 2017; Topakian et al., 2019). Currently, treatment with rituximab in MuSK-MG has a level IV evidence according to the latest multicenter prospective review conducted by Hehir et al. (2017). Although randomized clinical trials still need to be performed, all the studies conducted so far consistently indicate rituximab’s long-lasting effectiveness and safety for MuSK-MG patients, and an increase of its use in current clinical practice. Considering the high rate of positive response, it would be advisable that rituximab treatment is considered also for those severe patients who require a high level of immunosuppression even if they do not meet the criteria of refractory disease.

## Author Contributions

MC designed, conceptualized, and drafted the review. IK contributed to editing the text and created the original images. AV conceptualized, drafted, and supervised the review.

## Conflict of Interest

The University of Oxford holds a patent for use of MuSK for antibody assays, licensed to Athena Diagnostics. AV receives a proportion of royalties.

The remaining authors declare that the research was conducted in the absence of any commercial or financial relationships that could be construed as a potential conflict of interest.
